# 5. The PROTECT Trial: A Cluster Randomized Clinical Trial of Universal Decolonization with Chlorhexidine and Nasal Povidone Iodine Versus Standard of Care for Prevention of Infections and Hospital Readmissions among Nursing Home Residents

**DOI:** 10.1093/ofid/ofab466.005

**Published:** 2021-12-04

**Authors:** Loren G Miller, James A McKinnell, Raveena Singh, Gabrielle Gussin, Ken Kleinman, Raheeb Saavedra, Job Mendez, Tabitha D Catuna, James Felix, Justin Chang, Lauren Heim, Ryan Franco, Thomas Tjoa, Marlene Estevez, Brian Lewis, Julie Shimabukuro, Gregory Tchakalian, Aaron Minor, Crystal Torres, Kaye Evans, Cassiana Bittencourt, Jiayi He, Eunjung Lee, Christine Baesu, Julia Lu, Shalini Agrawal, Steven Park, Steven Tam, Shruti K Gohil, Philip A Robinson, Karl Steinberg, Nancy Beecham, Jocelyn Montgomery, DeAnn Walters, Nimalie D Stone, S G Sturdevant, Ellena M Peterson, Susan S Huang

**Affiliations:** 1 Harbor UCLA, Torrance, California; 2 LA BioMed at Harbor-UCLA Medical Center, Torrance, California; 3 Univeristy of California, Irvine, Irvine, California; 4 University of California, Irvine; 5 University of Massachusetts, Amherst, Massachusetts; 6 UC Irvine School of Medicine, Irvine, California; 7 Division of Infectious Diseases, the Lundquist Institute at Harbor-UCLA Medical Center, Torrance, CA, Torrance, California; 8 Lundquist Institute at Harbor-UCLA Medical Center, Torrance, California; 9 LA Biomed, Los Angeles, CA; 10 Soonchunhyang University Seoul Hospital, Seoul, Korea, Seoul, Seoul-t’ukpyolsi, Republic of Korea; 11 Hoag Hospital, Irvine, California; 12 Scripps Institute, Vista, CA; 13 California Association of Health Facilities, San Diego, CA; 14 CDC, Atlanta, GA; 15 NIH, Baltimore, Maryland

## Abstract

**Background:**

Nursing home (NH) residents are at high infection and hospital readmission risk. Colonization with multidrug-resistant organisms (MDROs) is common. In ICU and post-hospital discharge settings, decolonization has reduced infection rates. However, the effectiveness of this strategy in NHs is unclear.

**Methods:**

We performed a cluster randomized trial of 1:1 universal decolonization (decol) vs standard of care bathing (control) in 28 California NHs. After an 18 month baseline evaluation of hospitalization rates due to infection and MDRO prevalence, NHs were randomized to decol or control. Decol consisted of 1) chlorhexidine bathing; 2) nasal povidone iodine bid on admission x 5d and then M-F biweekly x 18 mo. Primary outcome was the probability that a transfer to a hospital was due to infection. Secondary outcome was the probability that a NH discharge was to a hospital.

**Results:**

Four of 28 NHs dropped from the trial (3 decol, 1 control). Mean facility baseline of hospital transfers due to infection was 58% and 57% in the control and decol groups. In the intervention period, proportions were 57% and 48% in the control and decol groups. When accounting for clustering within NHs, hospital transfers due to infection had an OR of 0.91 (95% CI: 0.82-1.02) in the control group and an OR of 0.73 (95% CI: 0.56-0.95) in the decol group when comparing intervention to baseline period. For the primary outcome, decol had a 18% greater impact v. control (P=0.005, Fig. A). Baseline proportion of NH discharges due to hospitalization was 37% and 39% in the control and decol groups. In the intervention period, proportions were 36% and 33%. When accounting for clustering within NHs, the proportion of discharges due to hospitalization had an OR of 1.14 (95% CI: 1.06-1.22) in the control group and 0.91 (CI: 0.77-1.07) in the decol group when comparing the intervention period to the baseline period. For the secondary outcome, decol had a 23% greater impact v. control (P< 0.0001, Fig. B).

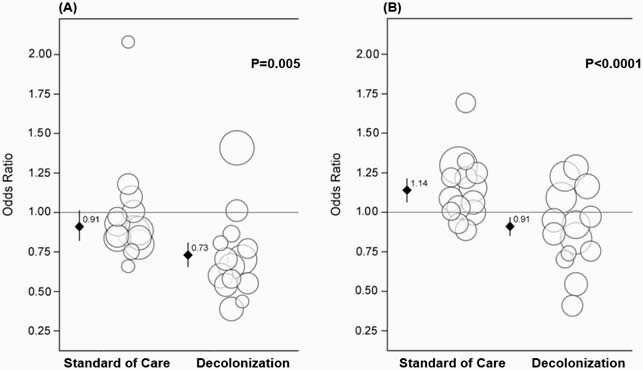

In this figure, each nursing home is represented by a circle. The size of the circle represents the amount of contributed patient days to the trial. The groups represent “as randomized” categories. Panel A) compares the probability that a transfer to a hospital was due to infection; panel B) compares the probability that a nursing home discharge was to a hospital. The y-axis represents the odds ratio of these probabilities comparing the baseline to the intervention period. The p values represent the significance of the difference between groups (the trial effect).

**Conclusion:**

Universal NH decolonization with chlorhexidine and nasal iodophor significantly reduced the proportion of transfers to hospitals due to infection and discharges due to hospitalization. Our findings suggest that NH decolonization reduces serious infections and can decrease morbidity in this vulnerable population.

**Disclosures:**

**Loren G. Miller, MD, MPH**, **Medline** (Grant/Research Support, Other Financial or Material Support, Contributed product) **Stryker** (Other Financial or Material Support, Contributed product) **Xttrium** (Other Financial or Material Support, Contributed product) **James A. McKinnell, MD**, **Medline** (Grant/Research Support) **Raveena Singh, MA**, **Medline** (Other Financial or Material Support, Conducted studies in which participating hospitals and nursing homes received contributed antiseptic and cleaning products) **Stryker (Sage**) (Other Financial or Material Support, Conducted studies in which participating hospitals and nursing homes received contributed antiseptic products) **Xttrium** (Other Financial or Material Support, Conducted studies in which participating hospitals and nursing homes received contributed antiseptic products) **Gabrielle Gussin, MS**, **Medline** (Other Financial or Material Support, Conducted studies in which participating hospitals and nursing homes received contributed antiseptic and cleaning products) **Stryker (Sage**) (Other Financial or Material Support, Conducted studies in which participating hospitals and nursing homes received contributed antiseptic products) **Xttrium** (Other Financial or Material Support, Conducted studies in which participating hospitals and nursing homes received contributed antiseptic products) **Ken Kleinman, PhD**, **Medline** (Other Financial or Material Support, Conducted studies in which participating hospitals received contributed antiseptic products) **Molnlycke** (Other Financial or Material Support, Conducted studies in which participating hospitals received contributed antiseptic products) **Raheeb Saavedra, AS**, **Medline** (Other Financial or Material Support, Conducted studies in which participating hospitals and nursing homes received contributed antiseptic and cleaning products) **Stryker (Sage**) (Other Financial or Material Support, Conducted studies in which participating hospitals and nursing homes received contributed antiseptic products) **Xttrium** (Other Financial or Material Support, Conducted studies in which participating hospitals and nursing homes received contributed antiseptic products) **Lauren Heim, MPH**, **Medline** (Other Financial or Material Support, Conducted clinical trials and studies in which participating hospitals and nursing homes received contributed antiseptic and cleaning products) **Molnlycke** (Other Financial or Material Support, Conducted studies in which participating hospitals received contributed antiseptic product) **Stryker (Sage**) (Other Financial or Material Support, Conducted clinical trials and studies in which participating hospitals and nursing homes received contributed antiseptic product) **Xttrium** (Other Financial or Material Support, Conducted clinical trials and studies in which participating hospitals and nursing homes received contributed antiseptic product) **Shruti K. Gohil, MD, MPH**, **Medline** (Other Financial or Material Support, Co-Investigator in studies in which participating hospitals and nursing homes received contributed antiseptic and cleaning products) **Molnycke** (Other Financial or Material Support, Co-Investigator in studies in which participating hospitals and nursing homes received contributed antiseptic and cleaning products) **Stryker (Sage**) (Other Financial or Material Support, Co-Investigator in studies in which participating hospitals and nursing homes received contributed antiseptic and cleaning products) **Susan S. Huang, MD, MPH**, **Medline** (Other Financial or Material Support, Conducted studies in which participating hospitals and nursing homes received contributed antiseptic and cleaning products) **Molnlycke** (Other Financial or Material Support, Conducted studies in which participating hospitals and nursing homes received contributed antiseptic and cleaning products) **Stryker (Sage**) (Other Financial or Material Support, Conducted studies in which participating hospitals and nursing homes received contributed antiseptic and cleaning products)**Xttrium** (Other Financial or Material Support, Conducted studies in which participating hospitals and nursing homes received contributed antiseptic and cleaning products)

